# Recommendations on the use of recombinant activated factor VII as an adjunctive treatment for massive bleeding – a European perspective

**DOI:** 10.1186/cc5026

**Published:** 2006-08-18

**Authors:** Jean-Louis Vincent, Rolf Rossaint, Bruno Riou, Yves Ozier, David Zideman, Donat R Spahn

**Affiliations:** 1 Department of Intensive Care, Erasme Hospital, Free University of Brussels, Route de Lennik 808, 1070 Brussels, Belgium; 2 Department of Anaesthesiology of the University Hospital, RWTH, Pauwelsstrasse 30, 52074 Aachen, Germany; 3 Department of Emergency Medicine and Surgery and Department of Anesthesiology and Critical Care, Hôpital Pitié-Salpêtrière, Assistance Publique-Hôpitaux de Paris, Université Pierre et Marie Curie, 47–83 Boulevard de l'Hôpital, 75651 Paris, France; 4 Service d'Anesthésie-Réanimation Chirurgicale, Hôpital Cochin, Assistance publique-Hôpitaux de Paris, Faculté de Medecine, Paris-V, Université Rene-Descartes, 27 Rue du faubourg Saint-Jacques, 75679 Paris, France; 5 European Resuscitation Council, ERC Secretariat, Universiteitsplein 1, 2610 Antwerp, Belgium; 6 Department of Anaesthesiology, University Hospital Lausanne, Rue du Bugnon 46, 1011 Lausanne, Switzerland

## Abstract

**Introduction:**

Our aim was to develop consensus guidelines for use of recombinant activated factor VII (rFVIIa) in massive hemorrhage.

**Methods:**

A guidelines committee derived the recommendations using clinical trial and case series data identified through searches of available databases. Guidelines were graded on a scale of A to E (with A being the highest) according to the strength of evidence available. Consensus was sought among the committee members for each recommendation.

**Results:**

A recommendation for the use of rFVIIa in blunt trauma was made (grade B). rFVIIa might also be beneficial in post-partum hemorrhage (grade E), uncontrolled bleeding in surgical patients (grade E), and bleeding after cardiac surgery (grade D). rFVIIa could not be recommended for use in the following: in penetrating trauma (grade B); prophylactically in elective surgery (grade A) or liver surgery (grade B); or in bleeding episodes in patients with Child–Pugh A cirrhosis (grade B). Efficacy of rFVIIa was considered uncertain in bleeding episodes in patients with Child–Pugh B and C cirrhosis (grade C). Monitoring of rFVIIa efficacy should be performed visually and by assessment of transfusion requirements (grade E), while thromboembolic adverse events are a cause for concern. rFVIIa should not be administered to patients considered unsalvageable by the treating medical team.

**Conclusion:**

There is a rationale for using rFVIIa to treat massive bleeding in certain indications, but only adjunctively to the surgical control of bleeding once conventional therapies have failed. Lack of data from randomized, controlled clinical trials, and possible publication bias of the case series data, limits the strength of the recommendations that can be made.

## Introduction

This study is endorsed by the European Society of Anaesthesiology (ESA), the European Society of Intensive Care Medicine (ESICM), the European Society for Emergency Medicine (EuSEM), the European Resuscitation Council (ERC), the European Haematology Association (EHA) and the European Association of Trauma and Emergency Surgery (EATES).

Uncontrolled massive hemorrhage is an important cause of morbidity and mortality. In patients with traumatic injury, it is second only to injuries to the central nervous system as the most common cause of death in the prehospital setting [1,2], and is the primary cause of early in-hospital (first 48 hours) mortality due to trauma [3]. In patients with liver disease, severe upper gastrointestinal (UGI) bleeding is fatal in about 30% of cases [4], whereas in patients undergoing open heart surgery, coagulopathic bleeding has been shown to increase both morbidity and mortality [5].

Massive hemorrhage is often characterized by a surgical or vascular component and a coagulopathic component. The surgical/vascular component can be corrected by surgical intervention or embolization. However, coagulopathic bleeding is more difficult to control. Coagulopathy arises through several interrelated mechanisms, which include the consumption of coagulation factors and platelets through repeated attempts to form clots during massive hemorrhage, the dilution of coagulation factors as a result of fluid resuscitation, and metabolic disorders (hypothermia or acidosis), which can affect the coagulation process [6]. Together with acidosis and hypothermia, coagulopathy forms the so-called 'lethal triad' in trauma, because of associated high mortality rates [7].

Conventional treatment options for coagulopathic/diffuse bleeding include fluid replacement (crystalloids and colloids) to maintain circulating volume, and the use of blood products such as red blood cells (RBCs), fresh frozen plasma (FFP), cryoprecipitate or fibrinogen, and platelets to replace the blood components lost during hemorrhage. However, attempts at resuscitation with large volumes of intravenous fluids can lead to an exacerbation of coagulopathy and may fail to arrest bleeding [8]. Furthermore, the use of blood products is associated with an increase in the risk of infections [9] and complications such as multiple organ failure and acute respiratory distress syndrome [10], which may result in increased mortality and morbidity [11,12].

The limitations of replacement therapy suggest the need for additional approaches to the treatment of coagulopathic bleeding. Hemostatic agents offer some promise as adjunctive therapy to be used with current treatments, but there are limited clinical trial data. Recombinant activated factor VII (rFVIIa, NovoSeven^®^; Novo Nordisk, Copenhagen, Denmark) may be useful in the treatment of coagulopathic bleeding. However, rFVIIa is currently approved worldwide only for the treatment of bleeding in patients with hemophilia A or B with inhibitors to coagulation factors VIII or IX [13]. In Europe, it is also approved for factor VII deficiency and Glanzmann's thrombasthenia in patients who are refractory to platelet transfusions, but it is not currently approved as an adjunctive treatment for massive or coagulopathic bleeding in any country.

In cases of injury, tissue factor (TF) is brought into contact with naturally occurring FVIIa, which is normally present in minute quantities, to initiate the coagulation pathway [14,15]. At pharmacological, supraphysiological doses, rFVIIa is able to bind to activated platelets at the site of injury and activate factors IX and X directly, leading to a thrombin burst [16]. As platelets are activated only at sites of TF exposure, it is believed that the action of rFVIIa is therefore localized to these sites. Nevertheless, a primary concern of treatment with rFVIIa is the possibility of an increased incidence of thrombotic adverse events, arising from a systemic activation of the coagulation pathway or from TF exposure at sites not associated with tissue injury, such as unstable coronary plaques [17].

rFVIIa is increasingly being used on a compassionate use basis [18]. However, there is no clear guidance on which patients are suitable for treatment, the appropriate timing of rFVIIa administration, and the most appropriate dose of rFVIIa to use. Although there are several ongoing Phase III clinical trials (see Additional file 1), the results will not be available for several years. Guidelines might therefore help to ensure that physicians receive appropriate guidance on the use of rFVIIa. This might be important when considering the potential costs, safety concerns and the risk of unpredictable adverse events, as well as the risks of overuse or inappropriate use associated with this treatment. In addition, published guidelines may help to protect physicians from the suggestions of substandard care when opting not to use rFVIIa in massive bleeding because of the lack of formally approved indications, and may also offer help with reimbursement when linked to the appropriate use of drugs not currently available on the open market.

This article presents a systematic review of the use of rFVIIa in patients with major hemorrhage, together with key recommendations for use based on these data. It is based on a consensus developed by experts in the fields of critical care medicine, anesthesia and intensive care medicine, emergency medicine, trauma, and hematology, representing the major European organizations, and is intended to assist the practicing physician in the appropriate use of this product.

## Materials and methods

Suitable references to compile this guidelines publication were identified from Medline, EMBASE and the Cochrane reviews (1980 to 2005) by using the following search terms: recombinant activated factor VII, recombinant factor VIIa, recombinant FVIIa, rFVIIa, and NovoSeven^®^. These results were cross-referenced with the terms trauma, coagulopathy, haemorrhage/hemorrhage, uncontrolled bleeding, and surgery. Additional searches were performed on recent clinical studies, case series and review publications to identify potential references not identified via the electronic database search.

The committee process began in July 2005 with initial electronic communications regarding structure, content and scope of the guidelines. References identified through the literature search were also made available to the committee. A meeting was held in September 2005 to assess the literature and develop recommendations for treatment for each potential rFVIIa indication. Clinical trial evidence supporting each recommendation was graded on the basis of a modified Delphi methodology with categorization, according to the criteria described in Table 1[19]. Each clinical trial was graded according to the presence or absence of key elements such as concealed randomization, blinded outcome assessment, intention-to-treat analysis, and explicit definition of a primary outcome. A strict evidence-based methodology with a scoring system was not used. The goal was total consensus among the members of the committee, which was reached for all the recommendations.

After the meeting in September, refinement of the recommendations continued through electronic communications and telephone discussions. The document was finalized and approved in December 2005 by the committee and the relevant scientific societies.

## Results

### Indications for use

Massive bleeding is classically described as loss of 1 blood volume in 24 hours. In the context of these recommendations, we should consider greater loss of blood volumes, such as loss of 50% blood volume in less than 3 hours. Under these conditions, administration of blood products would precede the administration of rFVIIa.

#### General recommendations

*Recommendation 1. *Every attempt should be made to control bleeding by conventional means. rFVIIa should not replace and/or delay surgery or any other methods used to control the source of bleeding, such as angiography with embolization. **Grade E**

*Rationale 1. *rFVIIa will be effective only once sources of major bleeding (such as open blood vessels) have been closed. Once major bleeding from damaged vessels has been stopped, it may be helpful to induce coagulation in areas of diffuse bleeding and oozing.

*Recommendation 2. *Traditional use of blood products, including RBCs, platelets, FFP, and cryoprecipitate/fibrinogen, should not be replaced by rFVIIa. **Grade E**

*Rationale 2. *rFVIIa is not a first-line treatment for bleeding. The focus of treatment is still replacement therapy with blood products such as RBCs, FFP, platelets, and cryoprecipitate/fibrinogen. rFVIIa should be considered only if first-line treatment with a combination of blood products and surgical approaches fails to control bleeding. However, it should be remembered that for rFVIIa to promote coagulation, sufficient levels of platelets and fibrinogen are required.

*Recommendation 3. *Every effort should be made to reduce the effects of, or to achieve the correction of, factors that may interfere with coagulation, including hypothermia, severe acidosis, low hematocrit, and hypocalcemia. An attempt to reverse the effects of any anticoagulant therapy should be made when possible. **Grade E**

*Rationale 3. *Hypothermia and coagulopathy in trauma are currently poorly understood; in general, the greater the degree of hypothermia, the greater the risk of uncontrolled bleeding. When severe injury is associated with hypothermia and acidosis, mortality rates of up to 100% have been reported. The effects of hypothermia include altered platelet function, impaired coagulation factor function (a 1°C decrease in temperature is associated with a 10% decrease in function), enzyme inhibition, and fibrinolysis [20,21]. Body temperatures below 34°C compromise blood coagulation, but this has been observed only when coagulation tests (prothrombin time [PT] and activated partial thromboplastin time) are performed at the low temperatures seen in patients with hypothermia, and not when assessed at 37°C (as is routine practice for such tests). Although Meng and colleagues [22] have shown that correction of hypothermia is not necessary for the proper functioning of rFVIIa, body temperature should be restored to as near physiological levels as possible, because even small reductions in temperature can result in slower coagulation enzyme kinetics [23].

In addition, coagulation disorders are aggravated by acidosis, caused by inadequate tissue oxygen supply. Moreover, hypocalcemia is frequently present in severely injured patients [24] and may require the administration of intravenous calcium, followed by frequent assessment to control serum levels of ionized calcium.

*Recommendation 4. *If major bleeding persists despite the above steps, the use of rFVIIa should be considered. To ensure maximal rFVIIa efficacy, attempts should be made to achieve the following: platelets more than 50,000 × 10^9^/l; fibrinogen 0.5 to 1.0 g/l; pH ≥ 7.20; hematocrit more than 24%. **Grade E**

*Rationale 4. *rFVIIa acts on the patient's own clotting system. To ensure the formation of a stable clot, fibrinogen levels will need to be maintained [25,26]. Furthermore, at pharmacological (that is, supraphysiological) doses, rFVIIa triggers the thrombin burst through direct binding to activated platelets; sufficient platelets must therefore be available. A reduction in platelet count also leads to impaired thrombin generation [27]. A recent study has shown that a pH of less than 7.10 will substantially reduce rFVIIa activity [22].

*Recommendation 5. *Before administering rFVIIa, the patient, or the patient's next of kin, should be informed about the type of treatment that they are receiving. **Grade E**

*Rationale 5. *Because rFVIIa is not approved for any of the indications discussed in this publication, the patient's next of kin should be informed that rFVIIa is being used outside the currently approved indications (off-label use).

### Control of overt bleeding

#### Trauma

*Recommendation. *An initial dose of 200 μg/kg rFVIIa, followed by two doses of 100 μg/kg, administered at 1 and 3 hours after the first dose, may reduce RBC transfusion requirements, the need for massive transfusion, and the incidence of respiratory failure (acute respiratory distress syndrome) in patients with blunt trauma [28]. **Grade B**

*Recommendation. *The effects of rFVIIa in patients with penetrating trauma are uncertain, and no recommendations can be made for this indication. **Grade B**

*Rationale. *Several case studies and case series have shown that treatment with rFVIIa can be beneficial in the treatment of coagulopathic bleeding after trauma [29-31]. In a large US case series (*n *= 81) in patients with coagulopathic bleeding as a result of trauma and other causes, rFVIIa at doses of between 40 and 150 μg/kg was successfully used to stop bleeding in 75% of these cases [31]. A retrospective cohort analysis of 29 patients treated with rFVIIa (initial dose 40 μg/kg, with 52% of patients receiving a second dose) matched with historical controls demonstrated significant reductions in RBC, platelet and cryoprecipitate requirements in the rFVIIa group with no increase in complications and a comparable mortality rate [32].

Most recently, guidelines for rFVIIa use have been published, based on findings from a case series of 36 patients who received rFVIIa on a compassionate use basis in Israel [30]. Treatment with rFVIIa successfully stopped bleeding in 72% of cases, leading the authors to recommend an initial dose of 120 μg/kg (between 100 and 140 μg/kg), with a second dose if required. If bleeding continues, a third dose can be considered only if the patient's coagulation parameters are within an acceptable range. The authors recognize the lack of any supporting clinical trial data for these recommendations and suggest that their dosing recommendations be taken as advisory.

Definitive recommendations on dosing require evidence from prospective, randomized, controlled clinical trials, and until recently this level of evidence was not available. The recently completed multicenter, randomized, double-blind, placebo-controlled study by Boffard and colleagues [28] examined the efficacy of rFVIIa in patients with blunt or penetrating trauma. Patients were randomized to receive either three doses of rFVIIa (200, 100, and 100 μg/kg) or placebo after they had received six units of RBCs, and received the first dose of their assigned medication after transfusion of a further two units of RBCs (eight in total), followed by a second and third dose, 1 and 3 hours after the initial dose. Treatment with rFVIIa produced a significant reduction in the primary endpoint, RBC transfusion requirements (a surrogate for blood loss), and significantly reduced the need for massive transfusions (more than 20 units of RBCs [*post hoc *definition]) in patients with blunt trauma surviving for more than 48 hours, and also significantly reduced the incidence of acute respiratory distress syndrome in all patients with blunt trauma.

Further support for the dose regimen recommended here comes from pharmacokinetic modeling techniques, which have shown that the dose regimen for rFVIIa treatment used in these randomized trials is capable of providing adequate plasma levels of drug to support hemostasis [33]. However, it should also be pointed out that the target concentration (40 U/ml) chosen in this study was based only on previous *in vitro *studies and thus remains a matter of debate.

Therefore, although the 200 μg/kg dose should be recommended, because it is the dose used in the only randomized study available, it remains possible that lower doses might be as efficient. Further studies are needed to answer this important question. Moreover, if there is clinical evidence that coagulopathy has been corrected by the first dose(s), there is no evidence to support the systematic administration of subsequent doses.

Although it might be possible to discuss recommendations for patients with blunt trauma, no recommendations are possible for those with penetrating trauma. No significant effects were seen on RBC transfusion requirements in these patients, although trends towards reduced RBC requirements and fewer massive transfusions were observed. In contrast to blunt trauma, penetrating trauma may be more easily controlled through surgical methods, and the level of bleeding is often lower than in blunt trauma. In the patients with penetrating trauma in this study, the reduced level of bleeding might have decreased the power to detect reductions in blood loss, and this might explain the lack of a significant reduction in RBC requirements. The issue of how to select appropriate patients to assess the effects of rFVIIa in penetrating trauma will need to be addressed in future studies.

#### Liver disease

*Recommendation. *Based on the currently available evidence, rFVIIa should not be used in patients with Child–Pugh A cirrhosis. **Grade B**

*Recommendation. *In patients with Child–Pugh B and C cirrhosis, the efficacy of rFVIIa in patients with bleeding episodes (esophageal and UGI bleeding, and bleeding after percutaneous needle biopsy) is uncertain. **Grade C**

*Rationale. *A preliminary, single-center, dose-escalation study in nonbleeding patients with advanced liver disease showed that treatment with rFVIIa could normalize PT and might therefore be useful in the treatment of bleeding due to liver disease. Ten patients with abnormal PT values were given three successive dosages of rFVIIa (5, 20, and 80 μg/kg) over a 3-week period. The mean PT was transiently corrected to normal in all three dose groups [34].

A randomized, double-blind, placebo-controlled trial assessing the efficacy and safety of rFVIIa in 245 cirrhotic patients with variceal and nonvariceal UGI bleeding produced inconclusive results. Patients were randomized to receive eight doses of 100 μg/kg rFVIIa or placebo, in addition to pharmacological and endoscopic treatment. No overall effect of rFVIIa on the primary composite endpoint (failure to control UGI bleeding within 24 hours after first dose, or failure to prevent rebleeding between 24 hours and day 5, or death within 5 days) was observed, and no significant differences were observed in mortality. However, *post hoc *analyses in the subgroup of Child–Pugh B and C cirrhotic patients indicated that administration of rFVIIa may decrease the proportion of patients who have failed to control variceal bleeding [35].

#### Post-partum hemorrhage

*Recommendation. *rFVIIa may be considered as a treatment for life-threatening post-partum hemorrhage but should not be considered as a substitute for, nor should it delay, the performance of a life-saving procedure such as embolization or surgery, nor the transfer to a referring center. **Grade E**

*Rationale. *No randomized, controlled clinical studies investigating rFVIIa use in patients with post-partum hemorrhage have been performed. However, several individual case reports have demonstrated that rFVIIa may be an effective bleeding control in patients with severe post-partum hemorrhage [36-38]. A review of 13 cases of post-partum hemorrhage by Boehlen and colleagues [39] also demonstrates a positive effect on bleeding for doses from as low as 17.5 μg/kg to 120 μg/kg. A recent case series in 12 patients with severe life-threatening post-partum hemorrhage treated at a women's clinic in Helsinki over a 16-month period also showed positive effects on bleeding after treatment with rFVIIa. Doses ranging from 42 to 116 μg/kg were used and 11 out of 12 patients showed either a partial or a good response in terms of the reduction in RBC/FFP/platelet transfusion requirements [40]. However, these results should be interpreted with care, because of potentially serious publication bias resulting from the likelihood that only successful cases are reported.

#### Uncontrolled bleeding in surgical patients

*Recommendation. *There have been individual case reports of the successful use of rFVIIa when all other standard measures of bleeding control have failed. In the view of the panel, common sense would suggest that it might be prudent to consider rFVIIa for surgical bleeding if all other options have been considered. **Grade E**

*Rationale. *No prospective, randomized clinical studies examining rFVIIa use in uncontrolled bleeding in surgical patients have been published. Several case studies have documented successful control of bleeding after administration of rFVIIa (doses between 80 and 120 μg/kg) in surgical settings, although such case studies may be subject to serious publication bias [41-51]. In cases where surgical bleeding is not controlled by conventional means, common sense suggests that rFVIIa may be considered, despite the potential for publication bias.

### Cardiac surgery

*Recommendation. *rFVIIa may be beneficial in controlling postoperative bleeding after cardiac surgery. **Grade D**

*Rationale. *There are currently no data from prospective, randomized, placebo-controlled clinical trials examining the efficacy of rFVIIa in cardiac surgery, although there is currently one ongoing clinical study (see Additional file 1). Small case series and several case studies have reported the successful use of rFVIIa in cardiac surgery. Several larger case series and one small pilot, randomized, placebo-controlled clinical trial have also reported beneficial effects of rFVIIa administration. However, all case series and case studies reported here may be subject to serious potential publication bias.

In a small randomized, placebo-controlled study, 20 patients undergoing complex cardiac surgery were randomized to receive rFVIIa or placebo after cardiopulmonary bypass. There was a significant reduction in the risk of requiring allogeneic RBCs and coagulation products in patients receiving rFVIIa in comparison with patients who received placebo [52].

A case series comprising 51 patients treated for intractable hemorrhage after cardiac surgery at a single center, with propensity-score-matched historical control patients from the same center, showed that rFVIIa may be of benefit. Patients received either 2.4 mg (44 patients) or 4.8 mg (7 patients) of rFVIIa, with a second dose as required. Treatment with rFVIIa significantly reduced requirements for transfusion of RBCs and other blood products, and there was a marked and significant reduction in International Normalized Ratio, whereas a small reduction in partial thromboplastin time was adjudged to be clinically insignificant [53].

In a retrospective case series of 16 patients from a single center who received rFVIIa after cardiac surgery, a mean dose of 65 μg/kg rFVIIa (range 24 to 192 μg/kg) resulted in significant reductions in both the volume of bleeding and the requirements for RBCs and FFP [54]. Similarly, a retrospective case series in 24 patients from a single center with uncontrollable life-threatening bleeding showed that a single rFVIIa dose of 60 μg/kg rFVIIa stopped or reduced bleeding in 18 out of 24 patients, whereas 5 patients required more than one dose. A significant reduction in blood loss through chest drains was also reported [55].

A case series of 40 patients from a single center included 24 patients with bleeding after cardiac surgery. Patients received a 90 μg/kg rFVIIa dose, followed by a second dose 6 hours later, if required. Twelve cardiac surgery patients (50%) died within 4 hours of treatment, and the effects of treatment could not be determined. In the 12 surviving patients, there were significant reductions in RBC, FFP, platelet, and cryoprecipitate requirements. Six of these remaining 12 patients survived to discharge, the remaining 6 dying between 3 and 30 days after treatment [56].

A review of 20 cases of bleeding after cardiac bypass showed that single or multiple doses of rFVIIa between 30 and 120 μg/kg (mean dose 101 μg/kg) successfully restored hemostasis. In 14 patients (70%), rapid hemostasis was achieved after a single dose of rFVIIa (mean dose 57 μg/kg), whereas in the remaining 6 patients, gradual hemostasis was achieved after a mean of 3.4 doses (mean cumulative dose 225 μg/kg) [57].

In a retrospective cohort analysis of 24 patients treated with rFVIIa (median initial dose 60 μg/kg, 42% of the patients receiving a second dose and 8% a third dose), who were matched with historical controls, blood loss and transfusion requirements were significantly reduced in the period after rFVIIa administration, but not after 24 hours. The requirement for platelet concentrates was reduced in the rFVIIa group, but 6-month survival rates were not significantly different. No thromboembolic complications were noted [58].

### Prevention of bleeding

#### Elective surgery

*Recommendation. *Prophylactic administration of rFVIIa in elective surgical patients is currently not recommended. **Grade A**

*Rationale. *In a recent double-blind, randomized, placebo-controlled trial, rFVIIa administered prophylactically failed to control bleeding in patients with normal hemostasis undergoing surgical repair of major traumatic fracture of the pelvis or the pelvis and acetabulum who were expected to have a large volume of blood loss [59]. Patients received 90 μg/kg rFVIIa or placebo as add-on therapy at the time of the first skin incision, in addition to intraoperative salvaged RBCs. Treatment with rFVIIa had no significant effect on the total volume of perioperative blood loss, the primary outcome variable, between the rFVIIa and placebo groups. Furthermore, there were no significant differences between the two groups in any transfusion parameters, including the total volume of blood components, the number of patients requiring allogeneic blood components, or the total volume of fluids infused. However, there was a significant reduction in postoperative blood loss (rFVIIa, 240 ml; placebo, 370 ml; *p *= 0.022).

Conversely, in patients undergoing retropubic prostatectomy in a double-blind, randomized, placebo-controlled, dose-escalation trial, treatment with rFVIIa significantly reduced perioperative blood loss and the number of patients requiring transfusions [60]. This study, in 36 patients, showed that patients treated with either 20 or 40 μg/kg rFVIIa in the early operative phase experienced significantly reduced perioperative blood loss compared with those in the placebo group. Furthermore, no patients in the higher-dose group required transfusions, compared with 7 out of 12 placebo-treated patients. The odds ratio for receiving any blood product in patients treated with recombinant factor VIIa compared with control patients was 0 (95% confidence interval 0.00 to 0.33). However, although this study was randomized, double blind and placebo controlled, the authors were aware of whether the administered dose was 20 or 40 μg/kg.

The differences in the results observed in these two studies might have occurred for several reasons, such as the age of the patients enrolled in the two studies, and the type and location of the surgery undergone. In addition, the timing of the administration in the two studies might have influenced the outcome. Patients received rFVIIa before the first incision in the study by Raobaikady and colleagues [59] but much later in the procedure in the study by Friederich and colleagues [60]. Given the relatively short half-life of rFVIIa, this might account for some of the difference in efficacy observed in the two studies.

Although there might be some beneficial effect on blood loss, the committee felt that, on the balance of current evidence, prophylactic use is not recommended.

#### Liver surgery

*Recommendation. *Prophylactic administration of rFVIIa during orthotopic liver transplantation (OLT) or liver resection is not recommended. **Grade B**

*Rationale. *Several studies have examined the effect of rFVIIa in liver surgery. An early single-center safety study showed that patients receiving 80 μg/kg before OLT required fewer transfusions than matched historical controls [61], whereas in these same patients it was shown that rFVIIa enhanced thrombin generation in a localized, time-dependent manner and did not lead to systemic activation of coagulation or fibrinolysis [62].

A larger multicenter, placebo-controlled trial examined the effects of a single dose of rFVIIa (20, 40, or 80 μg/kg) administered immediately before surgery in 82 patients undergoing OLT as a result of chronic liver disease [63]. This study failed to show any effect on the primary endpoint, RBC transfusion requirements, between placebo-treated and rFVIIa-treated patients. There were also no significant differences between treatment groups in the requirement for other transfusion products, total blood loss, crystalloid and colloid replacement volume, and the requirement for other hemostatic drugs during the perioperative period.

A subsequent randomized, placebo-controlled study in 183 patients undergoing OLT as a result of cirrhosis (Child–Turcotte–Pugh class B or C) used higher rFVIIa doses (60 and 120 μg/kg) or placebo repeated every 2 hours perioperatively [64]. This study also failed to show any significant differences between placebo and rFVIIa in perioperative RBC transfusion requirements, the primary endpoint, although significantly more rFVIIa-treated patients avoided RBC transfusions than the placebo group (6 out of 62 in the 60 μg/kg group versus 0 out of 61 in the placebo group; *p *= 0.03). There were also no significant differences in the requirement for any other transfusion products, including FFP, platelet concentrate, and fibrinogen [64]. Furthermore, compared with placebo, rFVIIa failed to show any benefits on overall blood loss, the requirements for crystalloid or colloid replacement, or length of stay in hospital or on the intensive care unit.

In a randomized, placebo-controlled study, 204 noncirrhotic patients undergoing liver resection received 20 or 80 μg/kg rFVIIa [65]. No significant reduction was observed in RBC requirements, blood loss, or the number of patients transfused.

### Monitoring

*Recommendation. *No specific method is currently available to indicate the need for rFVIIa or to monitor its efficacy. Monitoring of rFVIIa efficacy should therefore be performed visually to assess the level of bleeding after rFVIIa administration, and by an assessment of the transfusion requirements after dosing. **Grade E**

*Rationale. *Current laboratory tests are unlikely to provide an accurate reflection of the condition of the patient because of the time required to obtain the results. In many cases, samples taken for analysis are rewarmed to 37°C before assay, and this, together with the use of buffer solutions in laboratory tests, fails to reflect the coagulation status of patients who are acidotic or hypothermic. PT has been used to monitor rFVIIa activity, but this measure often overestimates the effects of rFVIIa because of its high sensitivity. The most accurate measure for monitoring the efficacy of rFVIIa is therefore to assess bleeding visually. If the bleeding has stopped, no further rFVIIa administration is required. The authors of several case studies and case series have commented on the immediate visible effect on bleeding in some patients after rFVIIa administration [29,31].

### General

#### Contraindications

*Absolute. *rFVIIa should not be administered to patients who are unsalvageable according to the clinical evaluation of the medical team treating the patient.

*Relative. *The risk:benefit ratio should be assessed in patients with coronary artery syndrome and in those with a presence or history of thromboembolic events. Unstable coronary plaques present TF on their surface [17]. Treatment with rFVIIa may promote coagulation on these plaques, leading to acute complete coronary artery occlusion or myocardial infarction [66,67]. In addition, rFVIIa should not be administered to patients with hypersensitivity to mouse, hamster, or bovine proteins. As a consequence of the manufacturing process, rFVIIa may contain traces of hamster proteins (the host cell used to propagate the cloned DNA), mouse proteins (specifically, immunoglobulin G from immunoaffinity purification columns), and bovine proteins (from the cell culture media).

#### Safety

Karkouti and colleagues observed an increased frequency of acute renal failure in cardiac surgery patients receiving rFVIIa [53]. However, thromboembolic adverse events after rFVIIa administration cause the greatest concern, particularly in patients with a previous history of thromboembolic events. An increased incidence of thromboembolic adverse events may arise as a result of systemic activation of the coagulation pathway.

A recent systematic review of all published studies and case series detailing rFVIIa use in nonhemophilia patients with severe bleeding estimated that the incidence of thromboembolic events was between 1% and 2% [68]. Data from the US Food and Drug Administration Adverse Event Reporting System concerning reports of serious thromboembolic adverse events during approved and off-label use of rFVIIa between March 1999 and December 2004 record some 431 adverse events, of which 168 reports described thromboembolic events [69]. The authors state that reports to the US Food and Drug Administration often lacked sufficient information to evaluate potential dosage associations, and that analysis of the relationship between adverse events and rFVIIa was hindered by concomitant medications, pre-existing medical conditions and the confounding indication; the authors also note that randomized, controlled trials are needed to establish the safety of rFVIIa in patients without hemophilia. Many clinical trials have shown no increase in thromboembolic events with rFVIIa in comparison with placebo [28,59,60,64,65]. However, there is uncertainty about thromboembolic events in patients with risk factors for such events and in those with arterial disease, in whom atherosclerotic plaques may expose TF and lead to the activation of coagulation at sites other than the site of injury.

A nonsignificant increase in thromboembolic events was observed in a recent trial of rFVIIa in patients with intracerebral hemorrhage [70]. This trend was observed only in the subgroup of patients receiving the highest rFVIIa dose (160 μg/kg). Because patients with intracerebral hemorrhage have a low bleeding rate, the clearance and half-life of rFVIIa is not markedly decreased, as it is in trauma patients with severe bleeding. It is therefore possible that administration of a very high dose might have resulted in very high blood concentrations. Given that rFVIIa has demonstrated a good safety profile in other studies, this observation may suggest that high doses in patients without severe bleeding might induce some adverse events in either future trials and/or clinical use.

Few data are available concerning the potential interactions between rFVIIa and other drugs used to treat coagulopathy – for example, aprotinin and desmopressin. Although instances have been reported in trauma and cardiac surgery patients [58] with no noticeable complications, caution is needed because the number of cases reported is too low for any valid conclusions to be drawn. The committee considers that the administration of these drugs before treatment with rFVIIa should not be considered as a contraindication for rFVIIa administration in coagulopathic patients. Furthermore, if rFVIIa has already been administered, there is no reason to recommend the administration of other drugs to treat coagulopathy. However, if rFVIIa is administered in addition to other agents, the physician should monitor the patient closely for possible thromboembolic adverse events.

## Summary and future directions

**Figure 1:**
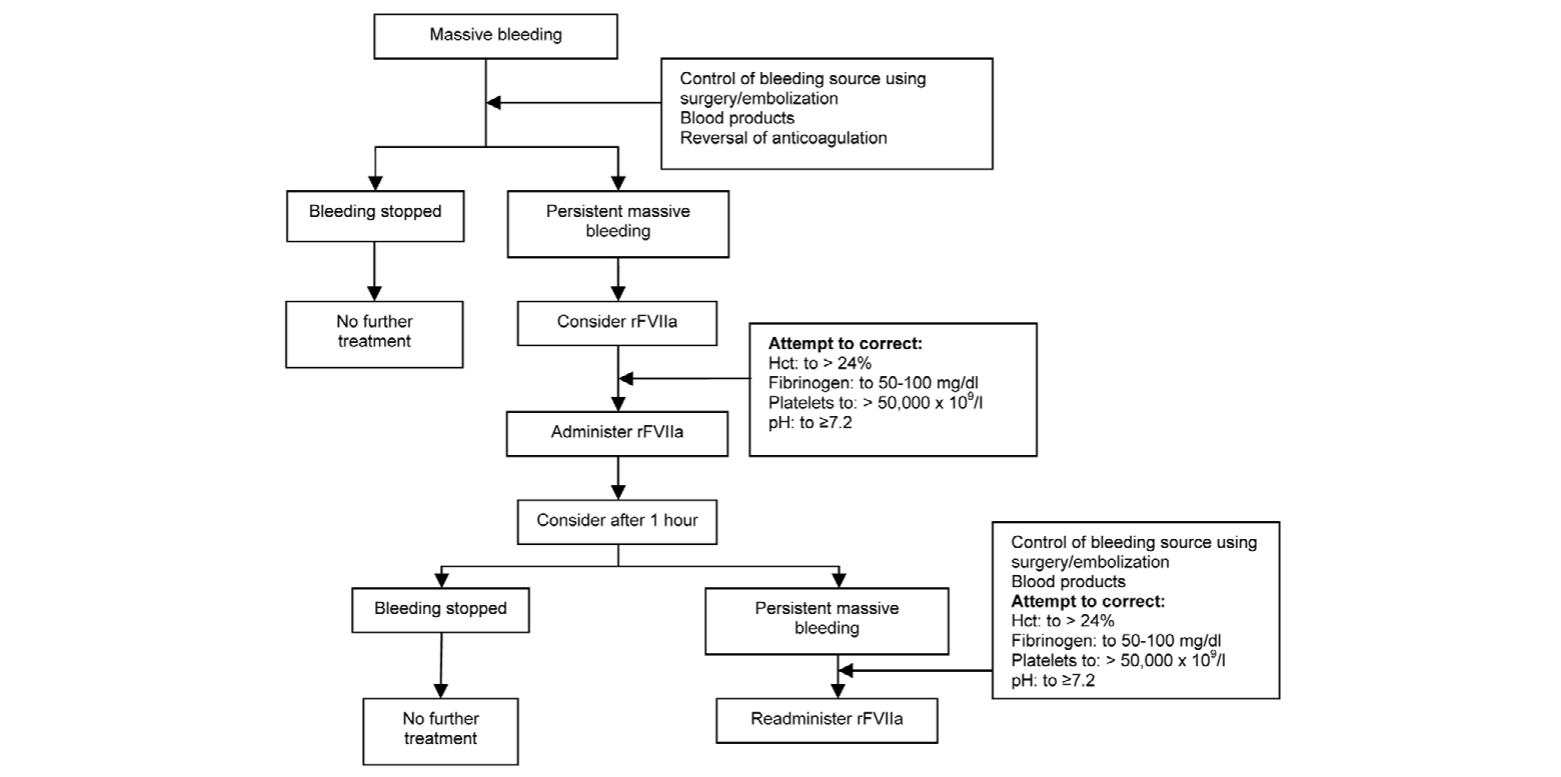
Algorithm for use of rFVIIa (see the text regarding rFVIIa dosing in different settings). Hct, hematocrit; rFVIIa, recombinant activated factor VII.

The purpose of these guidelines is to summarize the current evidence supporting the use of rFVIIa in the treatment of uncontrolled hemorrhage, and offer some guidance on appropriate use (see Figure 1). Some countries have developed local guidelines or consensus recommendations (for example, the recently published consensus recommendations on off-label use of rFVIIa by a US panel [71]), but European Union-wide standardized guidance (appropriate dose, timing of treatment, appropriate patients) is clearly needed. From the evidence presented from case series and clinical studies examining a wide variety of doses in patients with coagulopathy resulting from a range of causes, it is possible to draw some conclusions. However, it must be remembered that there is a potential for significant publication bias in many of the reported case studies and case series used to derive the recommendations presented here.

First, there is a rationale for the use of rFVIIa to treat uncontrolled hemorrhage in certain indications. However, rFVIIa should be used only as an adjunctive therapy to surgical control (and/or embolization), and only when all other attempts to control bleeding have failed. Second, treatment with rFVIIa seems to have some beneficial effect on blood loss and therefore on transfusion requirements. The risks of blood transfusions have been well documented [11] and should generally be avoided. In situations where transfusions are unavailable, or are judged to be too risky, treatment with rFVIIa is acceptable if death is the likely outcome of withholding rFVIIa treatment. Third, rFVIIa is generally well tolerated, with a good safety profile and relatively few thromboembolic events.

However, these positives should be balanced with some caveats. Although benefits have been observed with regard to blood loss and transfusion, there is currently no evidence to suggest that this effect translates to an improvement in morbidity or mortality. Furthermore, although current tolerability data are favorable, further study is required to establish whether certain patients are more at risk of thromboembolic (or other) adverse events, and which pre-existing risk factors need to be accounted for before administration. Finally, to make definitive recommendations for use, data from prospective, randomized, controlled, blinded clinical trials are required. Many of the data used to derive these recommendations come from large case series, which limits the strength of the recommendations that can be made. This is reflected in the grading score of each recommendation. The process of developing these guidelines and identifying where supporting evidence is weak has therefore identified future directions for research into rFVIIa, some of which are already being addressed by ongoing clinical trials.

## Conclusion

In response to a clinical need for practical guidance on the use of rFVIIa in the management of uncontrolled massive hemorrhage, consensus guidelines have been developed by an expert panel on the basis of a systematic review of the current evidence base. There is grade B evidence to support the use of rFVIIa, adjunctive to surgical control of bleeding, to manage bleeding due to blunt trauma, grade E evidence of a possible role for this therapy in the control of post-partum hemorrhage and grades E and D evidence, respectively, for the use of rFVIIa in the management of uncontrolled bleeding associated with surgery and cardiac surgery. At present, a paucity of data from randomized controlled trials with rFVIIa limits both the strength and the scope of clinical recommendations.

## Key messages

• Massive bleeding is an important cause of morbidity and mortality, and every attempt should be made to control bleeding by conventional means before considering a trial of rFVIIa.

• rFVIIa can be used adjunctive to surgery and the use of blood products to control bleeding in patients with blunt trauma (grade B); itmay be beneficial in controlling post-operative bleeding after cardiac surgery (grade D); it can be considered for the control of surgical bleeding if all other options have been considered (grade E), and it can be used as treatment for life-threatening post-partum hemorrhage but is not a substitute for life-saving surgery or embolization (grade E).

• rFVIIa is not currently recommended for use in the management of massive hemorrhage associated with penetrating trauma, elective surgery, liver surgery, bleeding due to Child–Pugh A, B, or C cirrhosis.

• rFVIIa efficacy should be monitored visually and by assessing transfusion requirements.

## Abbreviations

FFP = fresh frozen plasma; OLT = orthotopic liver transplantation; PT = prothrombin time; RBCs = red blood cells; rFVIIa = recombinant activated factor VII; TF = tissue factor; UGI = upper gastrointestinal.

## Competing interests

Meeting expenses and other financial support for the development of these guidelines were provided through an unrestricted educational grant from Novo Nordisk. No industry members served on the committee. Representatives from the sponsors were not permitted access to the committee responsible for developing these recommendations, nor were any of the sponsor's representatives present at the committee meeting or subsequent telephone conferences. No input into the guidelines development process by the sponsor was permitted, and the sponsors did not see the manuscript until it had been accepted for publication. JLV has received study grants and honoraria in the past from Novo Nordisk. RR has received lecture fees in the past from Novo Nordisk. BR received salaries and fees from Novo Nordisk in 2003 to 2005. YZ has received indirect departmental support from Novo Nordisk. DS is on the advisory board of Novo Nordisk and is a member of the ABC trauma faculty, which is managed by Thomson Physicians World GmbH and sponsored by an unrestricted grant from Novo Nordisk.

## Authors' contributions

The committee process began in July 2005 with initial electronic communications regarding structure, content and scope of the guidelines. References identified through the literature search were also made available to the committee. A meeting was held in September 2005 to assess the literature and develop recommendations for treatment for each potential rFVIIa indication. The goal was total consensus among the members of the committee, which was reached for all the recommendations. After the meeting in September, refinement of the recommendations continued through electronic communications and telephone discussions. The document was finalized and approved in December 2005 by the committee and the relevant scientific societies.

## Supplementary Material

Additional file 1 A Word file listing ongoing rFVIIa studies. Click here for file

## Figures and Tables

**Table 1:** Grading of recommendations and evidence.

Grading of recommendations	
A	Supported by at least two level I investigations
B	Supported by one level I investigation
C	Supported by level II investigations only
D	Supported by at least one level III investigation
E	Supported by level IV and V evidence

Grading of evidence	

I	Large randomized trials with clear-cut results; low risk of false-positive (alpha) error or false-negative (beta) error
II	Small randomized trials with uncertain results; moderate-to-high risk of false-positive (alpha) error and/or false-negative (beta) error
III	Nonrandomized, contemporaneous controls
IV	Nonrandomized, historical controls and expert opinion
V	Case series, uncontrolled studies, and expert opinion
