# Supplementary material for: Recommendations on the use of recombinant activated factor VII as an adjunctive treatment for massive bleeding – a European perspective
Source: Crit Care. 2006 Aug 18;10(4):R120. doi: 10.1186/cc5026 (PMC1750973; doi:10.1186/cc5026)
Supplement: Additional file 1 — A Word file listing ongoing rFVIIa studies. [file cc5026-S1.doc]

## Additional file 1

## Ongoing rFVIIa studies

| Indication | Phase | Description |
| --- | --- | --- |
| Traumatic injury | III | Multicentre, double-blind, randomized, parallel-group, placebo-controlled study. Treatment of refractory bleeding in trauma patients |
| UGI bleeding | II | Multicentre, double-blind, randomized, parallel-group, placebo-controlled study. Safety and efficacy in treatment of variceal bleeding in patients with cirrhosis |
| Cardiac surgery | II | Multicentre, double-blind, randomized, parallel-group, placebo-controlled dose-escalation study. Safety and efficacy in treatment of postoperative bleeding following cardiac surgery in patients requiring cardiopulmonary bypass |
| Cardiac surgery | III | Randomized, double-blind, placebo-controlled, parallel-group study. Safety and efficacy in salvage use following inadequate haemostatic response to conventional therapy in complex cardiac surgery |
| Surgery | II | Multicentre, double-blind, randomized, parallel-group, placebo-controlled comparison of rFVIIa and standard haemostatic replacement therapy following cardiac bypass surgery for paediatric congenital heart disease |
